# New perspective on sustained antidepressant effect: focus on neurexins regulating synaptic plasticity

**DOI:** 10.1038/s41420-024-01974-9

**Published:** 2024-05-01

**Authors:** Yuan Ruan, Ruolan Yuan, Jiaqi He, Yutong Jiang, Shifeng Chu, Naihong Chen

**Affiliations:** 1 Tianjin University of Traditional Chinese Medicine, Tianjin, PR China; 2 State Key Laboratory of Bioactive Substances and Functions of Natural Medicines, Institute of Materia Medica & Neuroscience Center, Chinese Academy of Medical Sciences and Peking Union Medical College, Beijing, PR China

**Keywords:** Long-term depression, Drug discovery, Emotion

## Abstract

Depression is highly prevalent globally, however, currently available medications face challenges such as low response rates and short duration of efficacy. Additionally, depression mostly accompany other psychiatric disorders, further progressing to major depressive disorder without long-term effective management. Thus, sustained antidepressant strategies are urgently needed. Recently, ketamine and psilocybin gained attention as potential sustained antidepressants. Review of recent studies highlights that synaptic plasticity changes as key events of downstream long-lasting changes in sustained antidepressant effect. This underscores the significance of synaptic plasticity in sustained antidepressant effect. Moreover, neurexins, key molecules involved in the regulation of synaptic plasticity, act as critical links between synaptic plasticity and sustained antidepressant effects, involving mechanisms including protein level, selective splicing, epigenetics, astrocytes, positional redistribution and protein structure. Based on the regulation of synaptic plasticity by neurexins, several drugs with potential for sustained antidepressant effect are also discussed. Focusing on neurexins in regulating synaptic plasticity promises much for further understanding underlying mechanisms of sustained antidepressant and the next step in new drug development. This research represents a highly promising future research direction.

## Facts

- Currently available medications for depression face challenges such as low response rates and short duration of efficacy. Additionally, many patients with depression also experience other psychiatric disorders, which further increases the risk of development if effectively managed overdue. Therefore, there is an urgent need for sustained antidepressant strategies.
- Ketamine and psilocybin gained attention as potential agents for sustained antidepressant effect. Review of recent studies highlights that synaptic plasticity changes as key events of downstream long-lasting changes in sustained antidepressant effect.
- Neurexins, which are key molecules involved in regulating synaptic plasticity, serve as a crucial link between synaptic plasticity and sustained antidepressant effect, involving mechanisms including protein level, selective splicing, epigenetics, astrocytes, positional redistribution and protein structure.
- Focusing on the role of neurexins in regulating synaptic plasticity holds great promise for understanding the underlying mechanisms and exploring new drugs for sustained antidepressant effect.

## Open questions

- Which of the many mechanisms by which neurexins modulate synaptic plasticity correspond specifically to those involved in sustained antidepressant effect?
- How do neurexins modulating synaptic plasticity relate upstream and downstream to existing antidepressant mechanisms?
- What are some of the drugs that potentially have modulated neurexins available to study sustained antidepressant effect?

## Introduction

Depression is one of the most serious psychiatric illness worldwide and is characterized by general and persistent low mood [1, 2]. Approximately 350 million people suffer from depression, seriously jeopardizing people’s physical and mental health [3]. Current research shows that the pathogenesis of depression is complicated and that the clinical manifestations caused by different pathogenic mechanisms [4, 5]. In addition, many depressed patients are accompanied by other mental illnesses such as schizophrenia and autism [6, 7]. If the illness is not treated promptly and effectively, many patients with mild depression gradually develop a major depressive disorder [8, 9]. This emphasizes the need and importance of long-lasting control in the treatment of both mild depression and major depressive disorder. Some classical antidepressants target on serotonin, norepinephrine, and dopamine are the predominant drugs used in current clinical practice [10]. Unfortunately, the classical antidepressants are not always effective for the different types of depression, and there are problems such as low response rates and delayed effects [11]. This is one of the main reasons why less than 40% of cases of depression are effectively treated in clinical practice and why the course and prognosis of the majority of patients are highly variable [12–14]. Against the backdrop of the need to address the problem of long-lasting control in the treatment of depression, the concept of sustained antidepressant effect was born [15].

Hallucinogens are the hot topic in research on sustained antidepressant effect [16]. Ketamine is currently the best-studied hallucinogen in the field of sustained antidepressant effect [17–20], and with psilocybin being granted breakthrough therapy for the treatment of depression by the U.S. Food and Drug Administration (FDA) in recent years [21], the concept of the sustained antidepressant has once again been promoted and renewed, and its attention has once again been taken to the next level [21–23]. Among these hypotheses about the mechanisms of sustained antidepressant effect, several studies have highlighted that the focus of the mechanism of sustained antidepressant pharmacodynamics should be centered on the downstream, long-term events of change induced by sustained antidepressant action. [14, 24]. This is truly compelling because in sustained antidepressant effect, long-lasting changing events and pharmacodynamic effects invariably correspond both in time and corresponding changes, emphasizing the need to focus on lasting changes downstream of events in sustained antidepressant effect. Numerous studies have observed changes in synaptic plasticity in the downstream long-lasting changes induced by hallucinogen intervention in depression [11, 16, 25], suggesting the importance of synaptic plasticity in sustained antidepressant effect.

**Fig. 1:**
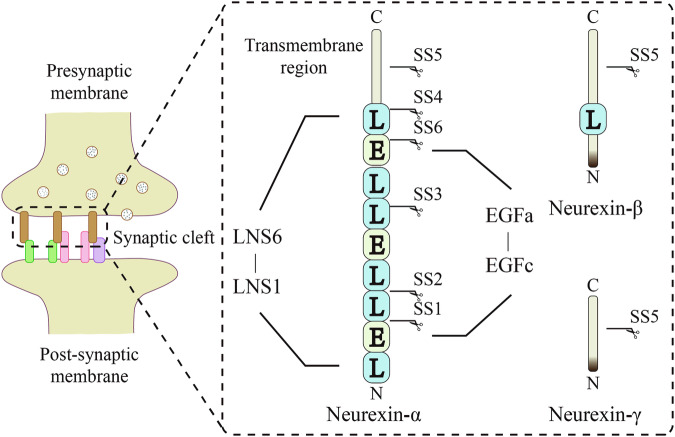
Schematic structure of the synaptic adhesion molecule neurexins. LNS laminin/neurexin/sex-hormone-binding globulin, SS1-SS6 selective splice sites 1–6, EGF epidermal growth factor-like domain.

Neurexins is a presynaptic adhesion molecule present in the synaptic cleft [26], the structure of which is shown in Fig. 1. In the synaptic cleft, neurexins bind to various postsynaptic adhesion molecules to form a variety of transsynaptic complexes [27], which are closely related to the formation and maintenance of synapses and form the basis of inter-synaptic information transfer [26]. Neurexins transsynaptic complexes play a central and important role in synaptic plasticity [26, 28], and the role of synaptic plasticity in sustained antidepressant effect has been widely reported [11, 16, 25]. This suggests the potential importance of neurexins transsynaptic complexes to sustained antidepressant effect. Therefore, this review generalizes and clarifies the important role of synaptic plasticity in sustained antidepressant effect. Subsequently, using neurexins as a starting point, the current research findings on the effects of neurexins transsynaptic complexes on synaptic plasticity are summarized and their use as a potential new perspective for sustained antidepressant effect is discussed.

## Synaptic plasticity participated in sustained antidepressant

Depression is a disorder with complex mechanisms characterized by a persistent low mood [4, 5]. A serious problem in current clinical practice is that depressed patients are usually accompanied by other types of psychiatric disorders [6, 7], which inevitably increases the risk of developing major depressive disorder and emphasizes the importance of timely and effective control of the disorder [8]. Nevertheless, the classic antidepressants used in clinical practice today are not always effective for the different types of depression and suffer from problems such as low response rates and delayed effects [11]. These problems result in less than 40% of depression cases receiving effective clinical treatment [12–14]. When studying the effect of sustained antidepressant effect, one cannot avoid the downstream long-lasting persistent events it causes, which is the key to sustained antidepressant effect [14, 24]. With this in mind, we summarized the downstream long-lasting sustained events of change in the last 5 years of studies on sustained antidepressant effect, with ketamine and psilocybin making up a large proportion of these studies. The relationship between synaptic plasticity and sustained antidepressant effect was compelling, as shown in Table 1.

**Table 1:** Ketamine and Psilocybin-induced downstream long-lasting sustained change events.

Drug	Model	Experimental objects	Downstream long-lasting sustained change events	Span	Ref
Ketamine	CUS	C57BL/6J mice	Depression-related behaviors; Dendritic spine density and dendritic spine morphology in prefrontal cortex (PFC), amygdala, and hippocampus	8 d	[29]
	Nrf2 KO	C57BL/6 mice	Depression-related behaviors; TrkB signaling pathway in PFC; synapse-associated proteins GluA1 and PSD-95 in PFC	7 d	[19]
	Anxiety disorders combined PTSD	Wistar Kyoto rat	Avoidance-related behaviors; Hippocampal synaptic plasticity	21 d	[31]
	Middle cerebral artery occlusion combined chronic unpredicted mild stress	Sprague-Dawley rat	Depression-related behaviors; NMDAR/CaMKII signaling pathway; Synaptic plasticity in the hippocampal dentate gyrus	7 d	[33]
	Chronic pain	C57BL/6J mice	Depression-related behaviors; T cell lymphoma invasion and metastasis 1 (Tiam1); Synaptic structure and functional plasticity in Anterior cingulate cortex (ACC) neurons	3 d	[32]
	Chronic CORT combined with repetitive restrain	C57BL/6J mice	Depression-related behaviors; Postsynaptic dendritic spines on prefrontal projection neurons	21 d	[30]
	-	CA1 region of mice hippocampal slices	NMDA receptor ion channels; K^+^ channels; Postsynaptic neuronal population spike amplitudes	2 h	[18]
	-	Mice mesencephalic and human induced pluripotent stem cells-derived DA neurons	AMPAR/mTOR/BDNF signaling pathway; Synaptic structural plasticity	3 d	[36]
	-	Swiss mice	BDNF and GFAP protein expression in PFC; Neuroplasticity	30 d	[34]
	-	C57BL/6 mice	Depression-related behaviors; NRG1/ ErbB4 signaling in parvalbumin-expressing (PV) neurons in the PFC; Neuroplasticity	7 d	[35]
Ketamine and guanosine	-	Swiss mice	Depression related behaviors; Dendritic spine density, mTORC1-dependent mechanisms, and synapse-associated proteins in the DG of the ventral hippocampus and the PFC	1 d	[45]
Psilocybin	Major depressive disorder	Human	EEG θ power amplitude; Neuroplasticity	14 d	[38]
	-	Human	Resting-state functional connectivity; Emotions and brain plasticity	30 d	[37]
	-	C57BL/6 J mice	Stress related behaviors; Excitatory neurotransmission; synaptic rewiring	30 d	[39]
		Danish slaughter pig	Hippocampal synaptic vesicle protein 2 A (SV2A); Increased persistent synaptogenesis	7 d	[40]
Ketamine and scopolamine	-	C57BL/6 J mice	Depression related behaviors; Phosphorylation of methyl CpG-binding protein 2 (MeCP2); BDNF protein expression; Synaptic plasticity	7 d	[46]
Ketamine and (2 R,6 R)-HNK	-	C57BL/6 mice	Function of AMPARs in the nucleus ambiguus; Altered Synaptic Plasticity in Brain Regions Associated with Rewarding Behavior	7 d	[41]
(2 R,6 R)-HNK	Learned helplessness (LH)	Sprague-Dawley rats	Depression-related behaviors; Glutamatergic transmission and surface GluR1 expression in the ventrolateral periaqueductal gray (vlPAG); Frequency and amplitude of miniature excitatory postsynaptic currents (mEPSCs) in the vlPAG	21 d	[43]
	-	midbrain dopaminergic neurons	Dendrite outgrowth	3 d	[44]
	-	C57BL/6J mice	BDNF/TrkB/mTORC1 signaling in the PFC; Synaptic function	5 d	[42]

There are a number of different model-based mechanistic studies in the reports on ketamine-related persistent antidepressants. Krzystyniak, A [29] and Qu, Y [19] respectively conducted studies on depression using chronic unpredictable stress (CUS) and knockout of Nrf2 as models. They confirmed the long-lasting antidepressant effect of a single injection of ketamine and observed changes in synaptic plasticity resulting from persistent changes in dendritic spines and synapse-associated proteins. Similarly, ketamine has been shown to be effective in inducing long-lasting changes in synaptic plasticity in depression-like behaviors in several models, including chronic corticosterone (CORT) associated with repetitive restraint stress [30], anxiety disorders associated with post-traumatic stress disorder (PTSD) [31], chronic pain [32], and middle cerebral artery occlusion associated with chronic unanticipated mild stress [33]. Similar experimental results have also been observed with ketamine in normal animals [34, 35]. In vitro studies, Jang, G observed a sustained high level of field potentials in the CA1 region by treating hippocampal brain slices with ketamine in vitro, suggesting a long-lasting change in synaptic plasticity [18]. More evidence was provided in the experiments of Cavalleri, L, who found a marked improvement in ketamine-induced synaptic function using dopaminergic (DA) neurons from the mice mesencephalon and human induced pluripotent stem cells, providing visual in vitro evidence for the intervention of ketamine in altering synaptic plasticity [36].

Psilocybin, a drug approved by the FDA as a breakthrough therapy for the treatment of major depressive disorder, is another hot topic in the field of sustained antidepressant effect [21]. Its sustained antidepressant effect is well established in the literature [21–23], but unfortunately its chronotropic changes following administration and the mechanisms involved have not been as extensively studied as with ketamine. In these literatures, Barrett, FS found that after a single oral administration of psilocybin to healthy volunteers for 30 days, functional magnetic resonance imaging (fMRI) results showed that the number of significant functional connections remained high in the resting-state brain, providing collateral evidence for the effect of psilocybin on human brain plasticity [37]. Further complementary studies by Skosnik, PD demonstrated a doubling of the theta power amplitude of the electroencephalogram (EEG) in patients with major depressive disorder after a single oral dose of psilocybin for 14 days [38]. In addition, the induction of synaptic plasticity following psilocybin administration was demonstrated in C57BL/6J mice [39] and in Danish slaughter pigs [40].

In terms of sustained antidepressant efficacy and downstream long-lasting synaptic plasticity, there is also a body of important evidence for the ketamine metabolite (2R,6R)-hydroxynorephedrine (2R,6R-HNK). Study in mice [41, 42], rats [43], and in vitro [44] have shown that 2R,6R-HNK has a sustained antidepressant effect and alters long-lasting synaptic plasticity. Furthermore, several studies on ketamine in combination with other drugs have shown similar evidence of synaptic plasticity changes [45, 46]. Through these studies, we substantiate the importance of synaptic plasticity in sustained antidepressant effect and establish it as a fundamental starting point for deciphering the intricate workings of sustained antidepressant effect.

## Neurexins participated in regulating synaptic plasticity

Changes in synaptic plasticity are key downstream events in sustained antidepressant effect, and understanding their mechanisms is important for resolving sustained antidepressant effect. Synapse is a cup-shaped or spherical structure in which neurons contact and communicate with each other. It mainly consists of a presynaptic membrane, a synaptic cleft, and a postsynaptic membrane [47]. Within the synaptic structure, there are many presynaptic or postsynaptic adhesion molecules that are considered synaptic organizers. The presence of these adhesion molecules ensures the transmission of information between neurons and is also closely related to changes in synaptic plasticity [48]. In contrast to postsynaptic adhesion molecules, very few species have been found in the current identification of presynaptic adhesion molecules [47]. Among them, neurexins, which are localized in the presynapse, have received much attention from researchers due to their ability to bind to a variety of postsynaptic adhesion molecules to form multiple transsynaptic complexes involved in synaptic plasticity [48].

**Fig. 2:**
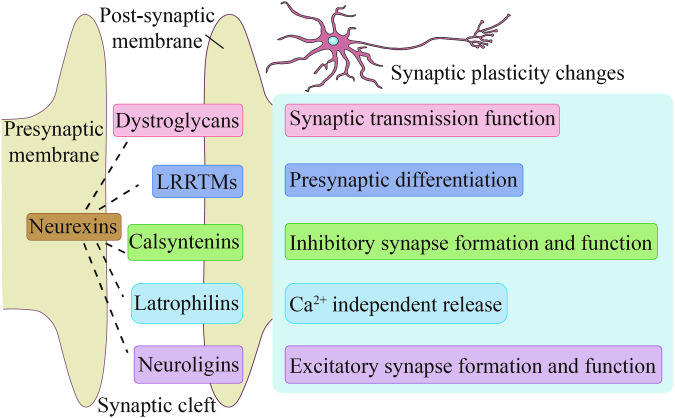
Postsynaptic adhesion molecules bound to neurexins and major functions.

Among the neurexins transsynaptic complexes in the mammalian brain, as shown in Fig. 1, there are three genetic phenotypes of the presynaptic adhesion molecule neurexins (neurexin-1, 2 and 3), with two isoforms of the longer neurexin-α and the shorter neurexin-β for each genotype, in addition to a specific γ- isoform of neurexin-1 [49]. The longer neurexin-α contains six LNS structural domains (for laminin/neurexin/sex-hormone-binding globulin domains) interspersed with three EGF-like repeat sequences. The shorter neurexin-β is generated transcriptionally from an internal promoter in which it contains an LNS6 structural domain corresponding to the N-terminally truncated and specific neurexin-α, and neurexin-γ is generated transcriptionally from the internal promoter of the specific neurexin-1 gene [47]. The presence of these LNS structural domains provides binding sites for various postsynaptic adhesion molecules that are essential for the formation of neurexins transsynaptic complexes [49, 50]. To date, about 50 genes for postsynaptic adhesion molecules have been identified, and many more are still unspecific and uncharacterized [48]. Among these postsynaptic adhesion molecules, some have been clearly identified that bind to neurexins, such as dystroglycans, LRRTMs, calsyntenins, latrophilins and neuroligins [26, 28], which are listed in Fig. 2. There are a number of recent studies on neurexins and the transsynaptic complexes formed by neurexins that relate to synaptic plasticity.

For the current study of neurexins in the context of synaptic plasticity, an interesting point is that neurexins per se are not required for synapse formation, and that deletion of neurexins does not affect the formation of most synapses [48, 51–53]. Rather, the formation of transsynaptic complexes with postsynaptic adhesion molecules is crucial for neurexins, which in turn modulate synaptic function and ultimately influence changes in synaptic plasticity [47]. Another interesting aspect that should be further investigated is the fact that in most studies on synaptic plasticity, changes in neurexins are always accompanied by changes in postsynaptic adhesion molecules. For example, changes in both the presynaptic adhesion molecule neurexins and the postsynaptic adhesion molecule neuroligins have been observed to be associated with changes in synaptic plasticity in early life stress, stressed-sedentary, Parkinson’s disease, and Alzheimer’s disease [54–58]. Similarly, an experiment on the overexpression of neuroligin 1 confirmed the association between changes in the ratio of two synaptic adhesion molecules, neuroligins and neurexins, and synaptic plasticity [59]. Similar evidence has been found for the formation of transsynaptic complexes between neurexins and other postsynaptic adhesion molecules. For example, the transsynaptic complexes of dystroglycans and neurexins correlate with the function of synaptic transmission [60], neurexins-LRRTMs transsynaptic complexes regulate presynaptic differentiation [61], neurexins-calsyntenins transsynaptic complexes correlate with inhibitory synapse formation and function [62] and neurexins-latrophilins transsynaptic complexes are associated with synaptic excitatory mechanisms Ca^2+^ independent release [63]. The evidence from these studies convinces us that neurexins are central regulators of synaptic properties, and we can clearly conclude that neurexins and neurexins transsynaptic complexes are intimately involved in synaptic plasticity.

## Neurexins participation in sustained antidepressant effect

Neurexins have been found to form different neurexins transsynaptic complexes by binding to different postsynaptic adhesion molecules via different gene regulation, selective splicing, and different LNS structural domains for their participation in regulating synaptic plasticity [48, 64, 65]. This suggests that modulating neurexins can alter the composition of neurexins transsynaptic complexes and hence intervene in synaptic plasticity. One important role for changes in synaptic plasticity is its involvement in sustained antidepressant effect. This prompted us to postulate that sustained antidepressant effect, which is intimately associated with synaptic plasticity, appears to have all the potential for future connections if changes in synaptic plasticity can be controlled longitudinally by manipulating neurexins. In order to demonstrate the broad potential of modifying neurexins as a strategy for sustained antidepressant effect, we examine the regulatory mechanisms of neurexins and their biological role in sustained antidepressant effect in this section.

### Potential mechanisms of neurexins participation in sustained antidepressant effect

#### Regulating protein expression levels of neurexins

**Fig. 3:**
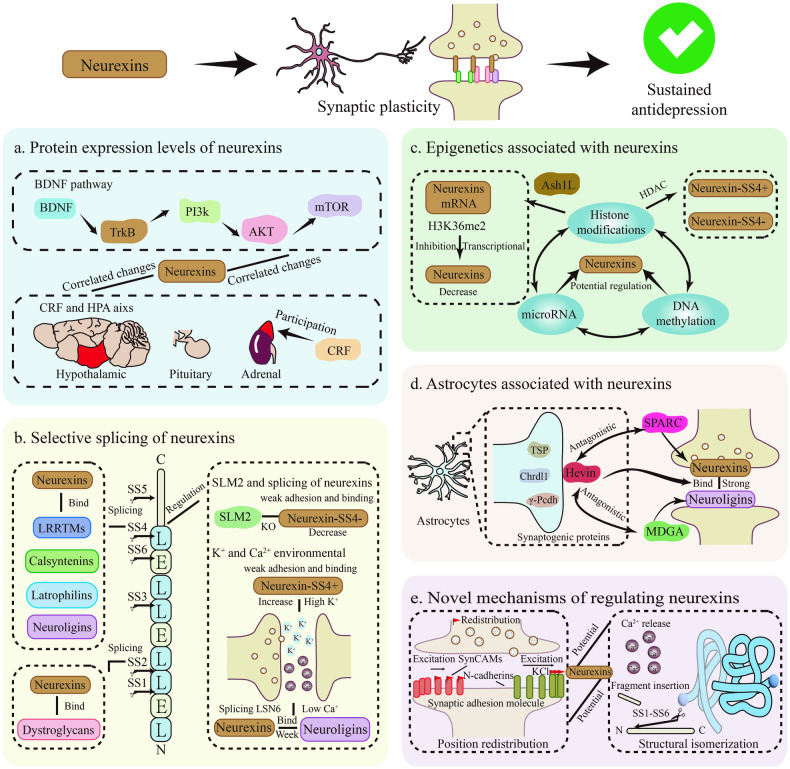
Potential mechanisms of neurexins participation in sustained antidepressant effect. **a** Regulating protein expression levels of neurexins. **b** Regulating selective splicing of neurexins. **c** Regulating epigenetics associated with neurexins. **d** Regulating astrocytes associated with neurexins. **e** Novel mechanisms of regulating neurexins. CRF corticotropin-releasing factor, HPA hypothalamic-pituitary-adrenal, SS1-SS6 selective splice sites 1–6, LNS laminin/neurexin/sex-hormone-binding globulin, SLM2 Sam68-like mammalian proteins 2, HDAC histone deacetylase, TSP thrombospondins, Chrdl1 Chordin-like 1, γ-Pcdh γ-protocadherin, Hevin high endothelial venule protein, SPARC secreted protein acidic and rich in cysteine, MDGA MAM domain-containing GPI anchor.

It has long been known that changes in synaptic plasticity are related to the amount of certain synaptic adhesion molecules in a synapse and thus how that synapse develops, is maintained and eventually disappears. Neurexins, the synaptic adhesion molecules that serve as regulatory centers for synaptic properties, are also the subject of much research on changes in synaptic plasticity and protein levels (Fig. 3a).

Among the neurexins, there are three genotypes of neurexin-1, 2 and 3. The three genotypes are highly expressed in synapses before the formation of extensive synapses, with neurexin-1 and neurexin-3 widely distributed between neuronal synapses in the cortical plate, and neurexin-2 distributed between neuronal synapses in the cortical wall [66]. neurexin-1 expression level increases with age, neurexin-3 remains stable, and neurexin-2 expression level is higher than that of neurexin-1 and neurexin-2 at early ages [66]. The expression level of neurexin-1 increased with age, neurexin-3 remained stable, and neurexin-2 was higher than neurexin-1 and neurexin-3 at the early stage, but intermediate between neurexin-1 and neurexin-3 at the later stage [66]. The three genotypes also differ in their function in synaptic plasticity, with neurexin-1 and neurexin-3 promoting synapse formation while neurexin-2 restricting it [67]. Long-lasting modulation targeting different genotypes leads to different changes in synaptic plasticity [68], which could be an important strategy for sustained antidepressant effect.

An in-depth mechanistic study has shown that activation of the BDNF signaling pathway significantly increases protein levels of neurexin-β and neuroligin 1 and synaptic plasticity in the hippocampus, possibly involving TrkB/PI3k/AKT/mTOR in this process [54]. It is known that BDNF, an important neurotrophic factor, plays a crucial role in sustained antidepressant effect [46]. This provides an explanation for the involvement of neurexins proteins in the mechanism of sustained antidepressant effect mediated by BDNF signaling, but recent studies have not clarified their relative upstream and downstream relationship to BDNF signaling, and the mechanism of this regulation at the protein level awaits further clarification. In addition, antagonists of corticotropin-releasing factor 1 (CRF 1) have some antidepressant and anti-anxiety effects [69, 70], via mechanisms possibly related to the involvement of CRF 1 in the hypothalamic-pituitary-adrenal (HPA) axis [58]. Despite the lack of definitive proof for sustained antidepressant effect, several studies have indicated that CRF knockout mice display increased synaptic plasticity and elevated neurexin-1 protein levels [58]. If this mechanism can be harnessed for long-lasting modulation, then CRF 1 antagonists, serving as antidepressant, present new potential for application. Nevertheless, further verification is required through specific experiments.

In relation to the regulation of neurexins protein, there is a possibility to manipulate its own activity through targeting expression generation and elimination. For instance, when neural activity is enhanced, the synthesis of neurexin-1 is increased, leading to elevated expression [71]. Additionally, certain crucial enzymes involved in protein breakdown, such as matrix metalloproteinases and α/γ-secretase, play significant roles in both the generation and elimination of neurexins protein activity [72, 73]. It is highly likely that directing these hydrolase activities towards achieving sustained enhancement of synaptic plasticity could result in long-term modulation of neurexins protein levels, thereby offering a potential avenue for sustained antidepressant effect.

#### Regulating selective splicing of neurexins

We know that neurexins consist of six LNS structural domains (LNS1–LNS6) interspersed with three EGF-like repeats, and that there are six special selective splice sites (SS1–SS6) under this basic structure [47]. Neurexins mRNA are spliced at different splice sites to generate different mRNA transcripts, usually with insertions, deletions, and substitutions of amino acids in the encoded proteins, a process that has led to the formation of a variety of splice isoforms of neurexins [72].

Different splice variants of neurexins correspond to different active functions, and their own affinity for different postsynaptic adhesion molecules varies (Fig. 3b) [74]. Under certain circumstances, selective splicing of neurexins has been shown to be an important regulator of changes in synaptic plasticity. For example, exon 20 on the SS4 fragment of neurexins is a highly utilized splicing site in the mRNA of all neurexins. Neurexins-SS4+, a splicing variant containing exon 20 on the SS4 fragment, exhibits only weak adhesion and binding properties, and when exon 20 of SS4 is spliced, synapses exhibit strong adhesion and mutual binding [75, 76]. The phenomenon has been clearly validated in the chronic variable stress (CVS) model. Notably, neurexin-3-SS4+ selective splicing propensity was induced in the hippocampus after transient CVS, and the mechanism of the subsequent impact of this effect may be related to the restriction of fast receptor trafficking induced by postsynaptic AMPA receptor anchoring, which is closely associated with reduced synaptic plasticity [76]. In addition, other splice sites on neurexins besides the SS4 splice site have been reported in the literature to be spliced to varying degrees to generate different neurexins splice isoforms. However, the functions induced by these splice sites are not well defined and require further in-depth studies [77, 78]. Crucially, selective splicing of neurexins also involves changes in the affinity of postsynaptic adhesion molecules. For example, splicing at SS2 in neurexins modulates binding to dystroglycans, and splicing regulation at SS4 alters the affinity of neurexins for postsynaptic adhesion molecules such as neuroligins, dystroglycans, LRRTMs, calsyntenins, and latrophilins [72, 77, 79]. The different affinity of neurexins for postsynaptic adhesion molecules implies that different neurexins are reconstituted in the synaptic complex, leading to changes in synaptic plasticity.

Splicing at different sites can alter the functional activity of the neurexins themselves or the affinity of postsynaptic adhesion molecules, thereby causing changes in synaptic plasticity (Fig. 3b). Based on the effects of neurexins splicing on synaptic plasticity, it is possible to link sustained antidepressant effect. It is then particularly important to tap into the mechanisms of active regulation of this process and to control it in the long term stage. For the regulation of variable splicing of neurexins, Traunmüller, L showed that the RNA-binding proteins SLM1 and SLM2, which belong to the STAR (RNA signaling and activating factor) family, are expressed in a mutually exclusive manner but are not complementary in the regulation of the splice variants of neurexins. Elimination of SLM2 led to a decrease in the non-complementarity of neurexins-SS4- splice variants, with a corresponding loss of strong adhesion and reciprocal binding [68, 80]. In a more in-depth study by Traunmüller, L showed that correction of SLM2 exon defects rescued SLM2 knockout-induced synaptic plasticity and behavioral deficits [80]. This evidence seems to emphasize the particularly strong specificity of SLM2 in controlling alterations in synaptic plasticity and the potential of SLM2 as a major breakthrough point for sustained antidepressant effect. Furthermore, a study on neuronal cells in vitro has shown a certain level of high K^+^ environment triggers membrane depolarization, which in turn leads to a sustained expression of neurexin-1-SS4+ and a reduction in neuronal neurexin-1-SS4- expression, an effect that occurs in astrocytes and even in neuronal cell injury, which inevitably affects synaptic plasticity [81]. Although the conclusions from this study are limited to neuronal cells in vitro, the mechanism of selective splicing of SS4 by neurexins in a physiological setting needs to be investigated by experiments that truly mimic neuronal activity to clarify its biological significance [81]. However, it must be noted that this conclusion emphasizes the importance of changes in K^+^ levels in the extracellular environment of neurons [82]. There are also similar studies focusing on the nature of binding of neurexins and LRRTMs, the results of which support the existence of a Ca^2+^ dependence of binding [61], similar to K^+^, may be involved in the regulation of variable splicing of neurexins, affecting the LNS6 structural domain of neurexins and thus influencing binding to the postsynaptic adhesion molecule neuroligins [83]. These findings consistently emphasize the importance of changes in the concentrations of various ions in the extracellular environment of neurons, and it is possible that targeting the concentrations of ions in the environment to permanently regulate the splicing of neurexins and thereby interfere with synaptic plasticity could be one of the mechanisms of sustained antidepressant effect.

#### Regulating epigenetics associated with neurexins

Epigenetics is one of the hypotheses that neurexins modulate synaptic plasticity [82]. Epigenetics refers to long-lasting changes in gene expression that are regulated by transcriptional, post-transcriptional, intra- or post-translational mechanisms (such as DNA methylation, histone modifications, and microRNA regulation) that do not involve DNA sequence alteration and are usually characterized by stability and longevity [84]. A summary of neurexins and epigenetics can be found in Fig. 3c. In the study by Freire-Cobo, C, the authors explained the selective splicing changes of downstream neurexins by the function of the epigenetic histone deacetylase (HDAC), but imperfectly, the article does not include indicators of epigenetics, and this explanation needs further verification [76]. Detailed evidence is provided by the study of Zhu, Τ that when neurexin-1α is transcriptionally repressed and activated in neurons in vitro, it is enriched around its promoter by synthesizing zinc-finger protein (ZFP) to histone methyltransferase (Ash1L) and histone marker H3K36me2, respectively, and that the transcriptional repression of neurexin-1α disappears when Ash1L is turned off [85]. Ash1L and H3K36me2 are important process indicators of histone modification in epigenetics, providing direct evidence that epigenetic involvement in the regulation of neurexins intervenes in synaptic plasticity [85]. On the other hand, epigenetics is a stable and long-lasting event that is associated with sustained antidepressant efficacy. A number of publications have investigated depression based on epigenetics [86, 87]. From this perspective, could epigenetics serve as a bridge between sustained antidepressant efficacy and neurexins that modulate synaptic plasticity? Will it be possible to improve the efficacy of sustained antidepressant effect? Of course, there are still many unanswered questions about epigenetics and neurexins, such as the specific upstream and downstream relationships, the processes involved, and whether or not other synaptic adhesion molecules cooperate, which still need to be substantiated by a variety number of experiments and data.

#### Regulating astrocytes associated with neurexins

Previous studies have found that astrocyte contact with neurons can alter the localization of neurexins at synapses, while the expression of neurexins at dendrites can also provide negative feedback on the increase in synapses mediated by astrocyte contact [88]. This suggests that astrocytes can modulate synaptic receptivity by mediating signaling and dendritic localization of neurexins. Astrocytes can encapsulate more than 100,000 synapses, and their role in regulating synaptic plasticity has been elucidated in several papers [72, 89]. This has led to the idea that astrocytes are involved in the regulation of synaptic plasticity by neurexins.

As shown in Fig. 3d, some studies have focused on the ability of astrocytes to coat synapses and found that astrocytes secrete the synaptogenesis protein high endothelial venule protein (Hevin), which has a function in regulating synapses [89]. With regard to the synaptogenesis protein Hevin secreted by astrocytes, the results of Singh, SK showed that Hevin is able to establish glutamatergic synapses by bridging neurexin-1α and neuroligin-1β, which promotes the formation of excitatory synapses [90]. This was further supported by research conducted by Fan, S. Additionally, the Hevin-regulated neurexin-related mechanisms were further enhanced by the discovery of the secreted protein acidic and rich in cysteine (SPARC), which is reciprocally antagonistic to neurexins binding, and MAM domain-containing GPI anchor (MDGA), which is reciprocally antagonistic to neuroligins binding in the neurexins-neuroligins transsynaptic complex [91]. These considerations provide some evidence that astrocytes are involved in neurexins regulation of synaptic plasticity. As for astrocytes, their association with depression has also been reported [92]. Further linking the relationship between synaptic plasticity and sustained antidepressant effect suggest an important link between this regulatory mechanism and sustained antidepressant effect. However, we know that astrocytes secrete far more than Hevin as synaptogenic proteins, such as thrombospondins (TSP), Chordin-like 1 (Chrdl1), γ-protocadherin (γ-Pcdh), etc. mentioned in the literature [72, 93]. These synaptogenesis proteins have been more or less associated with synaptic plasticity, but the interplay between them, the profound mechanistic process involved in the regulation of synaptic plasticity by neurexins, and the possible associations with sustained antidepressant effect have not yet been elucidated in detail and need to be further reviewed and explored by researchers.

#### Novel mechanisms of regulating neurexins

For the neurexins that regulate synaptic plasticity, there are a number of other novel mechanistic hypotheses associated with sustained antidepressant effect (Fig. 3e). Although these mechanisms suffer from the problem that there are few relevant studies and insufficient evidence, we can still believe that these potential mechanisms are worth validating and exploring, and that they may hold the secret to sustained antidepressant effect.

The first point we have focused on is the positional redistribution of synaptic adhesion molecules. The synaptic cleft consists of structurally distinct subregions, and synaptic adhesion molecules can branch into different regions of the synaptic cleft [94]. Recently, using high-resolution microscopy, the postsynaptic adhesion molecule SynCAMs was observed to redistribute selectively and extensively from the edges of the synaptic cleft in response to a long-lasting enhancement of synaptic plasticity [72, 94]. Similar redistribution changes following KCl-stimulated transient synaptic excitation have also been observed for N-cadherins in the literature [95], suggesting that synaptic adhesion molecules undergo positional redistribution in response to changes in synaptic function, which has the potential to alter interactions between synaptic adhesion molecules [72]. This finding is intriguing, but the position-dependent redistribution of neurexins has not yet been investigated. Synaptic adhesion molecules are associated with changes in synaptic plasticity, and it remains to be seen whether neurexins, as the core of the transsynaptic complex, are redistributed either by themselves or by synaptic adhesion molecules to which they bind, and once the mechanism of redistribution is clarified, it will be of great interest to further link with sustained antidepressant effect.

Another interesting mechanism is that, synaptic adhesion molecules undergo structural changes as a result of synaptic activity, which can lead to altered binding patterns. For example, neurexin-1β and LRRTM4 bind in a Ca^2+^-dependent manner [61], which may suggest that synaptic adhesion molecules can sense synaptic activity (Ca^2+^ release) and thereby cause a change in protein structure to form a protein conformation for binding by both pre- and postsynaptic adhesion molecules, leading to the formation of transsynaptic adhesion molecules. It has also been proposed that synaptic adhesion molecules sense synaptic changes occurring through an (allo)steric mechanism to adjust their interactions. It has been hypothesized that this mechanism may involve changes in splicing inserts, similar to those at SS1 and SS6 sites in neurexin-1*α* [72, 96]. The changes in protein structure resulting from these mechanisms would inevitably lead to changes in the transsynaptic complexes of neurexins, and the resulting link to sustained antidepressant effect could be a new and innovative idea. Of course, these theories are still pure conjecture at present, and deeper experimental validation is needed to clarify and exploit this mechanism.

### Drugs associated with neurexins modulating synaptic plasticity

To understanding the potential of neurexin-modulated synaptic plasticity in sustained antidepressant effect, we further summarize the drugs that may be involved in the modulation of synaptic plasticity by neurexins to expand the drug pool and link depression in depth to unlock the potential that these drugs may have in antidepressant or sustained antidepressant effect.

In our summary, we found that the types of disorders treated in the pharmacodynamic studies of these drugs were all psychiatric. First, we noted the stress-sedentary model and the CSV model, which may induce depression-like behaviors. In the stress-sedentary model, researchers conducted pharmacodynamic assessments using endurance running, a nontraditional pharmacologic tool, and observed an ameliorative effect on neurexins-β and neuroligin 1 expression and synaptic plasticity [54]. Similarly, a study in which the effect of long-lasting exercise on depression resistance was observed emphasizes the potential application of sustained exercise for sustained antidepressant effect [97]. This in part provides data support for the positive effects of sustained exercise workouts in synaptic plasticity as well as lasting antidepressant effects, affirming the importance of sustained exercise. In another study on a CSV model, the combination of dihydrocaffeic acid (DHCA) and Malvidin-3-O-glucoside (Mal-gluc) was observed to have a corrective effect on CSV-induced expression and selective splicing of neurexins [76]. DHCA and Mal-gluc, as externalizing metabolites of bioactive dietary polyphenol compounds, have been associated with depression [98], and in addition to modulating neurexins and synaptic plasticity, studies on their further sustained antidepressant activity should follow. There are also numerous studies on other psychiatric disorders that are not directly related to depression. In Alzheimer’s disease, for example, vitamin B12 protects against the synaptic adhesion molecules PSD-95, neurexin-1 and neurolgins and restores synaptic plasticity [57]. In Parkinson’s disease, treatment with Allopregnanolon (Allo) eliminated abnormalities in the electrophysiological aspects of synapses and abnormalities in the levels of the synaptic adhesion molecules PSD-95, neurexin-1, and neuroligins [56]. There is also a potentially protective neuropeptide, apelin-13, which has been shown to play an important role in neurological impairments such as depression and anxiety [99]. In the context of its multiple modeling ameliorative effects, we have focused on the ameliorative effects also exerted on neurexin-1 and neuroligins in response to Parkinson’s and memory impairment, which in turn restore impairments synaptic plasticity [55, 100]. This key pharmacological activity forms the theoretical foundation for establishing further connections to sustained antidepressant effect.

In summary, the evidences for synaptic plasticity modulated by the above-mentioned drugs in neurexins are conclusive, and we believe that their potential for sustained antidepressant effect should be investigated. Although the mechanisms involved in their involvement are not clear at the moment, which are our immediate challenge to overcome.

### Novel sustained antidepressant effect drugs found based on neurexins modulating synaptic plasticity

In addition to the report mechanisms involved in neurexins modulation of synaptic that may have sustained antidepressant effect, it is also feasible to look for drugs from synaptic plasticity. Because some researchers may not have tested the mechanisms by which drugs modulate synaptic plasticity in relation to neurexins, and some drugs with potential for sustained antidepressant effect based on the modulation of synaptic plasticity by neurexins may have been overlooked. In this way, we have a broader spectrum which is more conducive to the search for drug with a potentially sustained antidepressant effect.

In the context of synaptic plasticity, priority is given to the currently hotly researched class of hallucinogens. Hallucinogens such as ketamine and psilocybin are known for their sustained antidepressant effects and alterations in synaptic plasticity [16, 18, 21]. Traditionally, hallucinogens can be categorized into classical hallucinogens and non-classical hallucinogens depending on whether they act on 5-HT2A or not. Classical hallucinogens include Psilocybin, lysergic acid diethylamide (LSD), mescaline, 2,5-dimethoxy-4-methylamphetamine (DOM), 2,5-dimethoxy-4-iodoamphetamine (DOI) and others [101]. Non-classical hallucinogens include ketamine, tetrahydrocannabinol (THC), 3,4-dioxomethylamphetamine (DOM), and so on [102]. The main focus of these hallucinogens regarding sustained antidepressant effect studies has been on ketamine and psilocybin. Although the mechanisms involved in the modulation of synaptic plasticity by neurexins remain to be verified, it is important to note that they have a clear effect on synaptic plasticity and are candidates with sustained antidepressant potential.

Furthermore, there have been instances of hallucinogenic Chinese medicines, such as *Lanmaoa asiatica* from Yunnan, China, referred to as *“lurid bolete”*, that should also be the subject of investigation [103, 104]. Chinese medicine has a very long history of treating depression [105], and as a traditional Chinese medicine, *Lanmaoa asiatica* has been shown to have hallucinogen-like effects [106, 107], which are very similar to those of hallucinogens. There are also plants widely distributed in tropical Asia and Oceania as traditional spices in the Myrtaceae family [108, 109], plants in the Solanaceae family such as *Datura stramonium*, *Brugmansia* and *Hyoscyamus niger* [110, 111], *Cannabis* in the Cannabaceae family [112], *Peperomia cactus* in the Cactaceae family [113], and *Lophophora williamsii* in the Cactus family [113]. All of them have been reported in the literature to have potential hallucinogenic effect, so do they have a similar sustained antidepressant effect? What is the possible material basis? Based on the regulation of synaptic plasticity by neurexins and the exploration of the sustained antidepressant effect and material basis, this is also a new direction to expand the library of alternative agents for sustained antidepressant effect.

## Conclusion and perspectives

In summary, we begin with the key role of changes in synaptic plasticity in sustained antidepressant effect, focus on synaptic plasticity, and followed by a discussion of the modulation of synaptic plasticity by neurexins to demonstrate the potential and feasibility of studies of sustained antidepressant effect through the modulation of synaptic plasticity by neurexins. Lastly, by summarizing the reported drugs involved in the regulation of synaptic plasticity by neurexins and the drugs that could modulate synaptic plasticity by neurexins, we expanded the pool of alternatives for a sustained antidepressant and provided a new direction for sustained antidepressant research.

## References

[1] Zhang NN, Zhang Y, Wang ZZ, Chen NH (2022). Connexin 43: insights into candidate pathological mechanisms of depression and its implications in antidepressant therapy. Acta Pharm Sin.

[2] Zuo C, Cao H, Feng F, Li G, Huang Y, Zhu L (2022). Repetitive transcranial magnetic stimulation exerts anti-inflammatory effects via modulating glial activation in mice with chronic unpredictable mild stress-induced depression. Int Immunopharmacol.

[3] Huang Y, Wang Y, Wang H, Liu Z, Yu X, Yan J (2019). Prevalence of mental disorders in China: a cross-sectional epidemiological study. Lancet Psychiatry.

[4] Du Preez A, Eum J, Eiben I, Eiben P, Zunszain PA, Pariante CM (2021). Do different types of stress differentially alter behavioural and neurobiological outcomes associated with depression in rodent models? A systematic review. Front Neuroendocrinol.

[5] Mcintyre RS (2016). Implementing treatment strategies for different types of depression. J Clin Psychiatry.

[6] Hadjkacem I, Ayadi H, Walha A, Moalla Y, Ghribi F (2013). [Comorbidity of depression with other psychiatric disorders in adolescents: about 77 cases]. Tunis Med.

[7] Ghirardi L, Kuja-Halkola R, Butwicka A, Martin J, Larsson H, D’onofrio BM (2021). Familial and genetic associations between autism spectrum disorder and other neurodevelopmental and psychiatric disorders. J Child Psychol Psychiatry.

[8] Hegerl U, Schönknecht P, Mergl R (2012). Are antidepressants useful in the treatment of minor depression: a critical update of the current literature. Curr Opin Psychiatry.

[9] Wang YT, Wang XL, Feng ST, Chen NH, Wang ZZ, Zhang Y (2021). Novel rapid-acting glutamatergic modulators: targeting the synaptic plasticity in depression. Pharm Res.

[10] Patil CR, Suryakant Gawli C, Bhatt S (2023). Targeting inflammatory pathways for treatment of the major depressive disorder. Drug Discov Today.

[11] Kim JW, Suzuki K, Kavalali ET, Monteggia LM (2023). Bridging rapid and sustained antidepressant effects of ketamine. Trends Mol Med.

[12] Demyttenaere K, Bruffaerts R, Posada-Villa J, Gasquet I, Kovess V, Lepine JP (2004). Prevalence, severity, and unmet need for treatment of mental disorders in the World Health Organization World Mental Health Surveys. JAMA..

[13] Cipriani A, Furukawa TA, Salanti G, Chaimani A, Atkinson LZ, Ogawa Y (2018). Comparative efficacy and acceptability of 21 antidepressant drugs for the acute treatment of adults with major depressive disorder: a systematic review and network meta-analysis. Focus.

[14] Lee S, Jeong J, Kwak Y, Park SK (2010). Depression research: where are we now?. Mol Brain.

[15] Ma H, Li JF, Qiao X, Zhang Y, Hou XJ, Chang HX (2023). Sigma-1 receptor activation mediates the sustained antidepressant effect of ketamine in mice via increasing BDNF levels. Acta Pharm Sin.

[16] Rivera-García MT, Cruz SL (2023). The resurgence of hallucinogen drugs in clinical research. Rev Investig Clin.

[17] Cai M, Zhu Y, Shanley MR, Morel C, Ku SM, Zhang H (2023). HCN channel inhibitor induces ketamine-like rapid and sustained antidepressant effects in chronic social defeat stress model. Neurobiol. Stress.

[18] Jang G, Maciver MB (2021). Ketamine produces a long-lasting enhancement of CA1 neuron excitability. Int J Mol Sci.

[19] Qu Y, Shan J, Wang S, Chang L, Pu Y, Wang X (2021). Rapid-acting and long-lasting antidepressant-like action of (R)-ketamine in Nrf2 knock-out mice: a role of TrkB signaling. Eur Arch Psychiatry Clin Neurosci.

[20] Wang YT, Zhang NN, Liu LJ, Jiang H, Hu D, Wang ZZ (2022). Glutamatergic receptor and neuroplasticity in depression: Implications for ketamine and rapastinel as the rapid-acting antidepressants. Biochem Biophys Res Commun.

[21] Belouin SJ, Averill LA, Henningfield JE, Xenakis SN, Donato I, Grob CS (2022). Policy considerations that support equitable access to responsible, accountable, safe, and ethical uses of psychedelic medicines. Neuropharmacology.

[22] Meccia J, Lopez J, Bagot RC (2023). Probing the antidepressant potential of psilocybin: integrating insight from human research and animal models towards an understanding of neural circuit mechanisms. Psychopharmacology.

[23] Raison CL, Sanacora G, Woolley J, Heinzerling K, Dunlop BW, Brown RT (2023). Single-dose psilocybin treatment for major depressive disorder: a randomized clinical trial. JAMA.

[24] Mill J, Petronis A (2007). Molecular studies of major depressive disorder: the epigenetic perspective. Mol Psychiatry.

[25] Vargas MV, Dunlap LE, Dong C, Carter SJ, Tombari RJ, Jami SA (2023). Psychedelics promote neuroplasticity through the activation of intracellular 5-HT2A receptors. Science.

[26] Connor SA, Siddiqui TJ (2023). Synapse organizers as molecular codes for synaptic plasticity. Trends Neurosci.

[27] Noborn F, Sterky FH (2023). Role of neurexin heparan sulfate in the molecular assembly of synapses - expanding the neurexin code?. FEBS J.

[28] Hart MP, Hobert O (2018). Neurexin controls plasticity of a mature, sexually dimorphic neuron. Nature.

[29] Krzystyniak A, Baczynska E, Magnowska M, Antoniuk S, Roszkowska M, Zareba-Koziol M (2019). Prophylactic ketamine treatment promotes resilience to chronic stress and accelerates recovery: correlation with changes in synaptic plasticity in the CA3 subregion of the hippocampus. Int J Mol Sci.

[30] Moda-Sava RN, Murdock MH, Parekh PK, Fetcho RN, Huang BS, Huynh TN (2019). Sustained rescue of prefrontal circuit dysfunction by antidepressant-induced spine formation. Science.

[31] Fortress AM, Smith IM, Pang KCH (2018). Ketamine facilitates extinction of avoidance behavior and enhances synaptic plasticity in a rat model of anxiety vulnerability: Implications for the pathophysiology and treatment of anxiety disorders. Neuropharmacology.

[32] Ru Q, Lu Y, Saifullah AB, Blanco FA, Yao C, Cata JP (2022). TIAM1-mediated synaptic plasticity underlies comorbid depression-like and ketamine antidepressant-like actions in chronic pain. J Clin Investig.

[33] Abdoulaye IA, Wu SS, Chibaatar E, Yu DF, Le K, Cao XJ (2021). Ketamine induces lasting antidepressant effects by modulating the NMDAR/CaMKII-mediated synaptic plasticity of the hippocampal dentate gyrus in depressive stroke model. Neural Plast.

[34] Viana GSB, Vale EMD, Araujo ARA, Coelho NC, Andrade SM, Costa ROD (2020). Rapid and long-lasting antidepressant-like effects of ketamine and their relationship with the expression of brain enzymes, BDNF, and astrocytes. Braz J Med Biol Res.

[35] Grieco SF, Qiao X, Johnston KG, Chen L, Nelson RR, Lai C (2021). Neuregulin signaling mediates the acute and sustained antidepressant effects of subanesthetic ketamine. Transl Psychiatry.

[36] Cavalleri L, Merlo Pich E, Millan MJ, Chiamulera C, Kunath T, Spano PF (2018). Ketamine enhances structural plasticity in mouse mesencephalic and human iPSC-derived dopaminergic neurons via AMPAR-driven BDNF and mTOR signaling. Mol Psychiatry.

[37] Barrett FS, Doss MK, Sepeda ND, Pekar JJ, Griffiths RR (2020). Emotions and brain function are altered up to one month after a single high dose of psilocybin. Sci Rep.

[38] Skosnik PD, Sloshower J, Safi-Aghdam H, Pathania S, Syed S, Pittman B (2023). Sub-acute effects of psilocybin on EEG correlates of neural plasticity in major depression: Relationship to symptoms. J. Psychopharmacol.

[39] Shao LX, Liao C, Gregg I, Davoudian PA, Savalia NK, Delagarza K (2021). Psilocybin induces rapid and persistent growth of dendritic spines in frontal cortex in vivo. Neuron.

[40] Raval NR, Johansen A, Donovan LL, Ros NF, Ozenne B, Hansen HD (2021). A single dose of psilocybin increases synaptic density and decreases 5-HT(2A) receptor density in the pig brain. Int J Mol Sci.

[41] Yao N, Skiteva O, Zhang X, Svenningsson P, Chergui K (2018). Ketamine and its metabolite (2R,6R)-hydroxynorketamine induce lasting alterations in glutamatergic synaptic plasticity in the mesolimbic circuit. Mol. Psychiatry.

[42] Fukumoto K, Fogaça MV, Liu RJ, Duman C, Kato T, Li XY (2019). Activity-dependent brain-derived neurotrophic factor signaling is required for the antidepressant actions of (2R,6R)-hydroxynorketamine. Proc Natl Acad Sci USA.

[43] Chou D, Peng HY, Lin TB, Lai CY, Hsieh MC, Wen YC (2018). (2R,6R)-hydroxynorketamine rescues chronic stress-induced depression-like behavior through its actions in the midbrain periaqueductal gray. Neuropharmacology.

[44] Collo G, Cavalleri L, Chiamulera C, Merlo Pich E (2018). (2R,6R)-hydroxynorketamine promotes dendrite outgrowth in human inducible pluripotent stem cell-derived neurons through AMPA receptor with timing and exposure compatible with ketamine infusion pharmacokinetics in humans. Neuroreport.

[45] Camargo A, Dalmagro AP, Delanogare E, Fraga DB, Wolin IaV, Zeni ALB (2022). Guanosine boosts the fast, but not sustained, antidepressant-like and pro-synaptogenic effects of ketamine by stimulating mTORC1-driven signaling pathway. Eur Neuropsychopharmacol.

[46] Kim JW, Autry AE, Na ES, Adachi M, Björkholm C, Kavalali ET (2021). Sustained effects of rapidly acting antidepressants require BDNF-dependent MeCP2 phosphorylation. Nat Neurosci.

[47] Südhof TC (2017). Synaptic neurexin complexes: a molecular code for the logic of neural circuits. Cell.

[48] Sclip A, Südhof TC (2023). Combinatorial expression of neurexins and LAR-type phosphotyrosine phosphatase receptors instructs assembly of a cerebellar circuit. Nat. Commun.

[49] Sterky FH, Trotter JH, Lee SJ, Recktenwald CV, Du X, Zhou B (2017). Carbonic anhydrase-related protein CA10 is an evolutionarily conserved pan-neurexin ligand. Proc Natl Acad Sci USA.

[50] Wilson SC, White KI, Zhou Q, Pfuetzner RA, Choi UB, Südhof TC (2019). Structures of neurexophilin-neurexin complexes reveal a regulatory mechanism of alternative splicing. EMBO J.

[51] Dudanova I, Tabuchi K, Rohlmann A, Südhof TC, Missler M (2007). Deletion of alpha-neurexins does not cause a major impairment of axonal pathfinding or synapse formation. J Comp Neurol.

[52] Südhof TC (2018). Towards an understanding of synapse formation. Neuron.

[53] Missler M, Zhang W, Rohlmann A, Kattenstroth G, Hammer RE, Gottmann K (2003). Alpha-neurexins couple Ca2+ channels to synaptic vesicle exocytosis. Nature.

[54] Fang ZH, Lee CH, Seo MK, Cho H, Lee JG, Lee BJ (2013). Effect of treadmill exercise on the BDNF-mediated pathway in the hippocampus of stressed rats. Neurosci. Res.

[55] Haghparast E, Sheibani V, Abbasnejad M, Esmaeili-Mahani S (2019). Apelin-13 attenuates motor impairments and prevents the changes in synaptic plasticity-related molecules in the striatum of Parkinsonism rats. Peptides.

[56] Sheibani V, Rajizadeh MA, Bejeshk MA, Haghparast E, Nozari M, Esmaeili-Mahani S (2022). The effects of neurosteroid allopregnanolone on synaptic dysfunction in the hippocampus in experimental parkinsonism rats: An electrophysiological and molecular study. Neuropeptides.

[57] Mehrdad J, Leila E, Emsehgol N (2023). The effect of vitamin B12 on synaptic plasticity of hippocampus in Alzheimer’s disease model rats. Int J. Neurosci.

[58] Wang XD, Rammes G, Kraev I, Wolf M, Liebl C, Scharf SH (2011). Forebrain CRF_1_ modulates early-life stress-programmed cognitive deficits. J Neurosci.

[59] Dahlhaus R, Hines RM, Eadie BD, Kannangara TS, Hines DJ, Brown CE (2010). Overexpression of the cell adhesion protein neuroligin-1 induces learning deficits and impairs synaptic plasticity by altering the ratio of excitation to inhibition in the hippocampus. Hippocampus.

[60] Trotter JH, Wang CY, Zhou P, Nakahara G, Südhof TC (2023). A combinatorial code of neurexin-3 alternative splicing controls inhibitory synapses via a trans-synaptic dystroglycan signaling loop. Nat. Commun.

[61] Roppongi RT, Dhume SH, Padmanabhan N, Silwal P, Zahra N, Karimi B (2020). LRRTMs Organize Synapses through Differential Engagement of Neurexin and PTPσ. Neuron.

[62] Um JW, Pramanik G, Ko JS, Song MY, Lee D, Kim H (2014). Calsyntenins function as synaptogenic adhesion molecules in concert with neurexins. Cell Rep.

[63] Deák F, Liu X, Khvotchev M, Li G, Kavalali ET, Sugita S (2009). Alpha-latrotoxin stimulates a novel pathway of Ca2+-dependent synaptic exocytosis independent of the classical synaptic fusion machinery. J Neurosci.

[64] Boucard AA, Chubykin AA, Comoletti D, Taylor P, Südhof TC (2005). A splice code for trans-synaptic cell adhesion mediated by binding of neuroligin 1 to alpha- and beta-neurexins. Neuron.

[65] Chih B, Gollan L, Scheiffele P (2006). Alternative splicing controls selective trans-synaptic interactions of the neuroligin-neurexin complex. Neuron.

[66] Harkin LF, Lindsay SJ, Xu Y, Alzu’bi A, Ferrara A, Gullon EA (2017). Neurexins 1-3 each have a distinct pattern of expression in the early developing human cerebral cortex. Cereb. Cortex.

[67] Lin PY, Chen LY, Jiang M, Trotter JH, Seigneur E, Südhof TC (2023). Neurexin-2: an inhibitory neurexin that restricts excitatory synapse formation in the hippocampus. Sci Adv.

[68] Traunmüller L, Bornmann C, Scheiffele P (2014). Alternative splicing coupled nonsense-mediated decay generates neuronal cell type-specific expression of SLM proteins. J Neurosci.

[69] Wróbel A, Serefko A, Szopa A, Rojek K, Poleszak E, Skalicka-Woźniak K (2017). Inhibition of the CRF(1) receptor influences the activity of antidepressant drugs in the forced swim test in rats. Naunyn Schmiedebergs Arch Pharmacol.

[70] Bourke CH, Glasper ER, Neigh GN (2014). SSRI or CRF antagonism partially ameliorate depressive-like behavior after adolescent social defeat. Behav Brain Res.

[71] Fu Y, Huang ZJ (2010). Differential dynamics and activity-dependent regulation of alpha- and beta-neurexins at developing GABAergic synapses. Proc Natl Acad Sci USA.

[72] Rudenko G (2017). Dynamic control of synaptic adhesion and organizing molecules in synaptic plasticity. Neural Plast.

[73] Bajor M, Kaczmarek L (2013). Proteolytic remodeling of the synaptic cell adhesion molecules (CAMs) by metzincins in synaptic plasticity. Neurochem Res.

[74] Gomez AM, Traunmüller L, Scheiffele P (2021). Neurexins: molecular codes for shaping neuronal synapses. Nat Rev Neurosci.

[75] Aoto J, Martinelli DC, Malenka RC, Tabuchi K, Südhof TC (2013). Presynaptic neurexin-3 alternative splicing trans-synaptically controls postsynaptic AMPA receptor trafficking. Cell.

[76] Freire-Cobo C, Wang J (2020). Dietary phytochemicals modulate experience-dependent changes in Neurexin gene expression and alternative splicing in mice after chronic variable stress exposure. Eur J Pharmacol.

[77] Reissner C, Runkel F, Missler M (2013). Neurexins. Genome Biol.

[78] Treutlein B, Gokce O, Quake SR, Südhof TC (2014). Cartography of neurexin alternative splicing mapped by single-molecule long-read mRNA sequencing. Proc Natl Acad Sci USA.

[79] Boucard AA, Ko J, Südhof TC (2012). High affinity neurexin binding to cell adhesion G-protein-coupled receptor CIRL1/latrophilin-1 produces an intercellular adhesion complex. J Biol Chem.

[80] Traunmüller L, Gomez AM, Nguyen TM, Scheiffele P (2016). Control of neuronal synapse specification by a highly dedicated alternative splicing program. Science.

[81] Liakath-Ali K, Südhof TC (2021). The perils of navigating activity-dependent alternative splicing of neurexins. Front Mol Neurosci.

[82] Baxter PS, Dando O, Hardingham GE (2023). Differential splicing choices made by neurons and astrocytes and their importance when investigating signal-dependent alternative splicing in neural cells. Front Mol Neurosci.

[83] Sheckler LR, Henry L, Sugita S, Südhof TC, Rudenko G (2006). Crystal structure of the second LNS/LG domain from neurexin 1alpha: Ca2+ binding and the effects of alternative splicing. J Biol Chem.

[84] Kuehner JN, Walia NR, Seong R, Li Y, Martinez-Feduchi P, Yao B (2023). Social defeat stress induces genome-wide 5mC and 5hmC alterations in the mouse brain. G3 (Bethesda).

[85] Zhu Τ, Liang C, Li D, Tian M, Liu S, Gao G (2016). Histone methyltransferase Ash1L mediates activity-dependent repression of neurexin-1α. Sci Rep.

[86] Meneses-San Juan D, Lamas M, Ramírez-Rodríguez GB (2023). Repetitive transcranial magnetic stimulation reduces depressive-like behaviors, modifies dendritic plasticity, and generates global epigenetic changes in the frontal cortex and hippocampus in a rodent model of chronic stress. Cells.

[87] Dalton VS, Kolshus E, Mcloughlin DM (2014). Epigenetics and depression: return of the repressed. J Affect Disord.

[88] Barker AJ, Koch SM, Reed J, Barres BA, Ullian EM (2008). Developmental control of synaptic receptivity. J. Neurosci.

[89] Allen NJ (2014). Astrocyte regulation of synaptic behavior. Annu Rev Cell Dev Biol.

[90] Singh SK, Stogsdill JA, Pulimood NS, Dingsdale H, Kim YH, Pilaz LJ (2016). Astrocytes assemble thalamocortical synapses by bridging NRX1α and NL1 via Hevin. Cell.

[91] Fan S, Gangwar SP, Machius M, Rudenko G (2021). Interplay between hevin, SPARC, and MDGAs: Modulators of neurexin-neuroligin transsynaptic bridges. Structure.

[92] Liao Y, Xing Q, Li Q, Zhang J, Pan R, Yuan Z (2021). Astrocytes in depression and Alzheimer’s disease. Front Med.

[93] Khaspekov LG, Frumkina LE (2023). Molecular Mechanisms of Astrocyte Involvement in Synaptogenesis and Brain Synaptic Plasticity. Biochemistry.

[94] Perez De Arce K, Schrod N, Metzbower SWR, Allgeyer E, Kong GK, Tang AH (2015). Topographic mapping of the synaptic cleft into adhesive nanodomains. Neuron..

[95] Yam PT, Pincus Z, Gupta GD, Bashkurov M, Charron F, Pelletier L (2013). N-cadherin relocalizes from the periphery to the center of the synapse after transient synaptic stimulation in hippocampal neurons. PLoS ONE.

[96] Chen F, Venugopal V, Murray B, Rudenko G (2011). The structure of neurexin 1α reveals features promoting a role as synaptic organizer. Structure.

[97] Jemni M, Zaman R, Carrick FR, Clarke ND, Marina M, Bottoms L (2023). Exercise improves depression through positive modulation of brain-derived neurotrophic factor (BDNF). A review based on 100 manuscripts over 20 years. Front Physiol.

[98] Wang J, Hodes GE, Zhang H, Zhang S, Zhao W, Golden SA (2018). Epigenetic modulation of inflammation and synaptic plasticity promotes resilience against stress in mice. Nat Commun.

[99] Lv SY, Chen WD, Wang YD (2020). The Apelin/APJ system in psychosis and neuropathy. Front Pharmacol.

[100] Gazmeh S, Azhir M, Elyasi L, Jahanshahi M, Nikmahzar E, Jameie SB (2022). Apelin-13 protects against memory impairment and neuronal loss, Induced by Scopolamine in male rats. Metab. Brain Dis.

[101] Nichols DE (2018). Chemistry and structure-activity relationships of psychedelics. Curr Top Behav Neurosci.

[102] Nichols DE (2016). Psychedelics. Pharmacol Rev.

[103] Wu G, Li Y-C, Zhu X-T, Zhao K, Han L-H, Cui Y-Y (2016). One hundred noteworthy boletes from China. Fungal Divers.

[104] Yang N, Zhang S, Zhou P, Zhang W, Luo X, Cao J (2022). Analysis of volatile flavor substances in the enzymatic hydrolysate of lanmaoa asiatica mushroom and its maillard reaction products based on E-nose and GC-IMS. Foods.

[105] Xia B, Chen C, Tao W (2021). Neuroplasticity: a key player in the antidepressant action of chinese herbal medicine. Am J Chin Med.

[106] Ma J, Xia J, Li HJ, Su LJ, Xue R, Jiang S (2023). Four cases of reported adverse effects from black boletoi, *Anthracoporus nigropurpureus* (Boletaceae) mushroom ingestion. Toxicon.

[107] Li H, Zhang Y, Liu Z, Zheng F, Zhao B, Wu G (2022). Species diversity of poisonous mushrooms causing poisoning incidents in Yunnan Province, Southwest China. Mycosystema..

[108] Mao C, Zhang F, Li X, Yang T, Zhao Q, Wu Y (2023). Complete chloroplast genome sequences of Myristicaceae species with the comparative chloroplast genomics and phylogenetic relationships among them. PLoS ONE.

[109] Barman R, Kumar Bora P, Saikia J, Konwar P, Sarkar A, Kemprai P (2024). Hypothetical biosynthetic pathways of pharmaceutically potential hallucinogenic metabolites in Myristicaceae, mechanistic convergence and co-evolutionary trends in plants and humans. Phytochemistry.

[110] Cunningham N (2008). Hallucinogenic plants of abuse. Emerg Med Australas.

[111] Kerchner A, Farkas A (2020). Worldwide poisoning potential of Brugmansia and Datura. Forensic Toxicol.

[112] Ibarra-Lecue I, Mollinedo-Gajate I, Meana JJ, Callado LF, Diez-Alarcia R, Urigüen L (2018). Chronic cannabis promotes pro-hallucinogenic signaling of 5-HT2A receptors through Akt/mTOR pathway. Neuropsychopharmacology.

[113] Watkins JL, Li Q, Yeaman S, Facchini PJ (2023). Elucidation of the mescaline biosynthetic pathway in peyote (Lophophora williamsii). Plant J.

